# Distribution of nursing workforce in the world using Gini coefficient

**DOI:** 10.1186/s12912-023-01313-w

**Published:** 2023-05-05

**Authors:** Erfan Kharazmi, Najmeh Bordbar, Shima Bordbar

**Affiliations:** Health Human Resources Research Centre, School of Management and Medical Information Sciences, Shiraz University of Medical Sciences, Shiraz, Iran

**Keywords:** Nursing workforce, Gini coefficient, HDI, World countries

## Abstract

**Introduction:**

Unequal Access to human resources for health, reduces access to healthcare services, worsens the quality of services and reduces health outcomes. This study aims to investigate the distribution of the nursing workforce around the world.

**Methods:**

This is a descriptive-analytical study, which was conducted in 2021. The number of nurses and world populations was gathered from World Health Organization (WHO) and The United Nations (UN) databases. The UN has divided world countries based on the Human Development Index (HDI) into four categories of very high, high, medium and low HDI. To investigate the distribution of the nurses around the world, we used the nurse population ratio (per 10,000 population), Gini coefficient, Lorenz curve and Pareto curve.

**Findings:**

On average, there were 38.6 nurses for every 10,000 people in the world. Nations with the very high HDI, had the highest nurse/population ratio (95/10,000), while the low HDI nations had the lowest nurse/population ratio (7/10,000). Most nurses around the world were females (76.91%) who were in the age group of 35–44 (29.1%). The Gini coefficient of nations in the each four HDI categories varied from 0.217 to 0.283. The Gini coefficient of the nations between the four HDI categories was 0.467, and the Gini coefficient of the whole world was 0.667.

**Conclusion:**

There were inequalities between countries all over the world. Policymakers should focus on the equitable distribution of the nursing workforce across all local, national and regional levels.

## Introduction

World Health Organization (WHO) defines Human Resources for Health as “all the people engaged in measures intending to improve health” [1]. Human resources as the heart of health in every society constitute the most significant and integral part of the healthcare system [2]. According to WHO, access to public health, Universal Health Coverage (UHC), and equal access to healthcare services depend on having a high-quality health workforce. Also, there is strong relationshipbetween the number of health workers and health outcomes, as the number of human resources for health has a significant impact on the achievement of Sustainable Development Goals (SDG), reduction of maternal mortality, infant mortality and under 5-year mortality rate [2–4].

On the other hand, in addition to quantity, proper distribution of health workers is essential to ensure fair access to health services [5]. Evidence suggests that the health human workforce ratio (the number of nurses or physicians per population), varies in different countries [2]. This anomaly denotes that investment in human resources for health is not only inadequate, but is also ineffective in its distribution [2]. In its 2006 health report, the WHO projected that over 4.3. million health workers were required to fill the positions of health personnel across the globe, with Africa taking a significant share with around 1.5 million people (35%) [1, 6]. Inequality in the distribution of human resources has also been confirmed in various studies in Poland, Mongolia, China, India, Sudan, Cameroon, and Brazil [5–10].

Moreover, nurses have accounted for the largest professional group of health providers across the world. Nurses play a pivotal role in the health system and provide a full scale of responsibilities to improve health, prevention, treatment and rehabilitation. There is a great variety of nursing workforce in different regions and countries and the shortage of nurses as well as other nursing workforce issues have always been a health service challenge worldwide [11, 12]. According to the State of the World’s Nursing 2020 (SWN), there are large-scale inequalities in the density and distribution of nurses at the world level in regions covered by the WHO and nations within those regions [4]. the minimum ratio of nurses to the population in Angola in the study year is equal to 0.012 per 10,000). The highest ratio is related to Monaco and is equal to 201 (per 10,000) while the average of the world is about 40 (per 10,000).According to these statistics, 114 countries are lower than the world’s average in terms of the ratio of nurses and 75 countries are higher than that (13).Since the healthcare workforce plays a vital role in the performance of the healthcare systems, and the general public health, inequality in access to human resources for health may reduce access to healthcare services, aggravates the quality of services, and eliminates health advantages [8]. As stated, the description, analysis and perception of inequality in the distribution of healthcare workforce are critical. Accordingly, monitoring changes in human resource inequalities in health care is essential to identify gaps, enable a better understanding of different countries and regions, and facilitate the implementation of effective and appropriate interventions. In sum, this study aimed to review the inequality of nursing workforce distribution across the world.

## Methods

This is a descriptive-analytical study which was developed in 2021. The number of nurses and world populations was gathered from WHO and UN databases. In each case, recorded data from the latest year, as confirmed by the WHO, was provided for calculations.

Accordingly, demographic data from countries and HDI index in 2020 as well as the number of nurses in 2018 and 2019 served as the basis of the calculations. When data in the main databases were lacking, other credible databases such as the websites of Health Ministries of those countries were used. If state data could not be provided from any sources, that specific country would be removed from the research population. Countries were classified based on the HDI index, consistent with the UN’s classification process in 2020. To calculate the HDI index, three factors of life expectancy, literacy level and Gross Domestic Product (GDP) are taken into account. The HDI index is a number between 0 and 1, and the larger this index, the more developed that nation. The UN has classified world countries into very high, high, medium and low HDI indices. The UN has delisted 6 countries due to their defective data, so included a final 189 countries in the classification. Sixty-six countries were included in the very high, 53 ones in the high, 37 ones in the medium and 33 ones in the low HDI groups [14].

To investigate the distribution of nursing workforce in the world, the Gini coefficient based on the Lorenz curve was applied. Gini coefficient is known as one of the most common distribution criteria and also as one of the top tools for measuring inequality [8]. This curve determines which group has the highest number of nurses.

This study calculates the Gini coefficient in three different states. The Gini coefficients of the same-group nations in the each of four-group category, between the four groups of nations and the Gini coefficient between all countries of the world were calculated. The Gini coefficient index represents the area ratio between the Lorenz curve and equality line to the whole region under the equality line. This index has a value ranging between 0 and 1, with 0 representing complete equality and 1 denoting complete inequality. If this index stands between 0.20 and 0.35, the distribution is relatively equal, between 0.35 and 0.50, the distribution is relatively unequal, and if between 0.50 and 0.70, the distribution is completely unequal [15]. To calculate the Gini coefficient, the following formula was used [16].

1 $$G=1-\sum _{i=0}^{n}\left({Y}_{i+1}+{Y}_{i}\right)\text{*}\left({X}_{i+1}+{X}_{i}\right)$$

n: Total number of groups.

Yi: Cumulative percentage of nurses in the in the group.

Xi: Cumulative percentage of the population in the group.

**Fig. 1:**
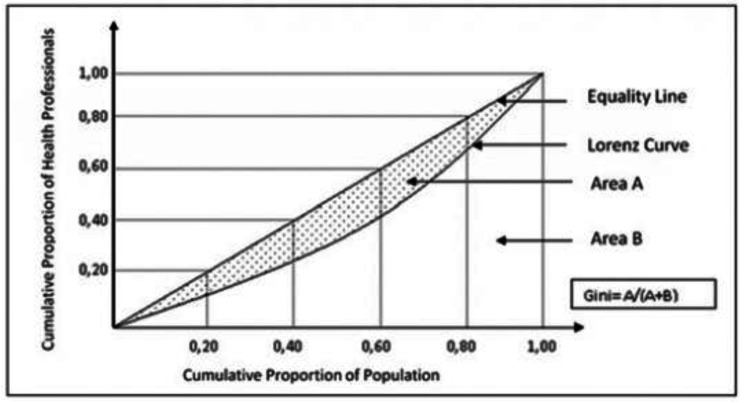
Lorenz curve and Gini coefficient (14)

In Lorenz curves, the horizontal axis is used to show the population of the countries and the vertical axis to show the number of nurses. Figure 1 shows the Lorenz curve and Gini coefficient calculation. For more consistency, all calculations were performed in Excel 2019 software. The ratio of nurses per 10,000 population was used to determine the distribution of nurses. The Pareto curve was also used to determine the cumulative distribution of the nurses in different nations using their populations.

### Findings

Results showed that Norway and Niger had the highest and lowest HDI indices of 0.957 and 0.394 in the world, respectively. In the meantime, 38.34% of the world’s population was in the high HDI group. Also, 58.22% of the world’s population had an HDI index of higher than the world’ average (0.682) (Table 1).

Overall, there are around 30 million nurses across the world, which accounted for an average rate of 38.6 nurses per 10,000 people. The highest and lowest distribution rate of nurses to the population were seen in very high HDI nations with around 95/10,000, and in low HDI nations with around 7/10,000. Besides, countries with high HDI rates had 33/10,000, while medium-HDI countries had 19/10,000. More than 50% of the world’s nurses were in very high HDI group countries (20% of the world’s population) and 2.3% of nurses were in low HDI group countries (12% of the world’s population). Most of the world’s nurses (76.91%) were females. The highest percentage of female nurses (87.44%) pertained to very high HDI nations, while the lowest percentage of female (55.03%) pertained to low HDI group nations. Most world nurses (29.1%) were in the age group of 35.44. Furthermore, the highest percentage of nurses over 64 years (9.72%) was in the very high HDI nations (Table 1).

**Table 1:** Distribution of nurses in different HDI group countries

Grouping factor		very high HDI	High HDI	Medium HDI	Low HDI	Ave / Sum
HDI	Max num.	0.957	0.796	0.697	0.546	Ave:0.682
	Average num.	0.879	0.747	0.618	0.487	
	Min num.	0.804	0.703	0.554	0.394	
World population	Max num.	332,915,073	1,444,216,107	1,393,409,038	211,400,708	Sum:7,800,775,176
	Average num.	24,224,955	58,641,460	64,283,712	29,515,895	
	Min num.	18,169	53,544	116,254	1,002,187	
	Total num.	1,574,622,110	2,990,714,471	2,269,929,924	965,508,671	
	%Of total	20.185	38.339	29.099	12.377	Sum:100
Nurses variables	Max num.	5,223,437	3,844,503	3,328,854	195,757	Sum:29,968,106
	Average num.	231,550	192,632	125,575	21,811	
	Min num.	132	199	41	571	
	Total num.	15,050,758	9,824,244	4,395,145	697,959	
	%Of total	50.223	32.782	14.666	2.329	Sum:100
	%Male	12.16	15.92	22.45	44.97	Ave:23.87
	%Female	87.44	84.07	81.105	55.03	Ave:76.91
	%Age:<25	0.45	2.4	3.68	2.98	Ave:2.3775
	%Age:25–34	23.84	26.36	37.7	27.05	Ave:28.7375
	%Age:35–44	23.16	30.03	28.38	34.81	Ave:29.095
	%Age:45–54	21.47	22.7	17.69	23.79	Ave:21.4125
	%Age:55–64	21.36	12.97	10.25	8.95	Ave:13.3825
	%Age:>64	9.72	5.54	2.3	2.42	Ave:4.995

**Fig. 2:**
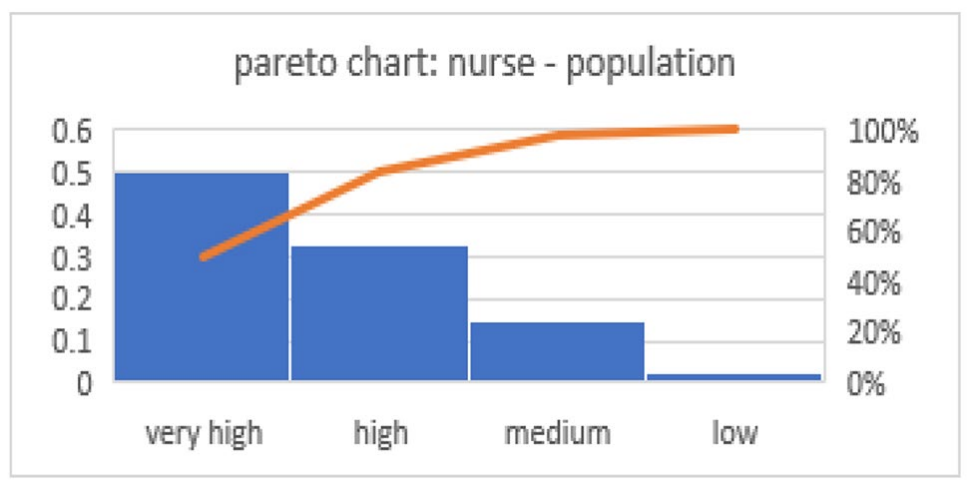
Pareto curve of nurse distribution to the population in 4 groups of countries

Pareto curve findings suggested that over 80% of the world’s nurses fell under very high and high HDI groups (Fig. 2).

**Fig. 3:**
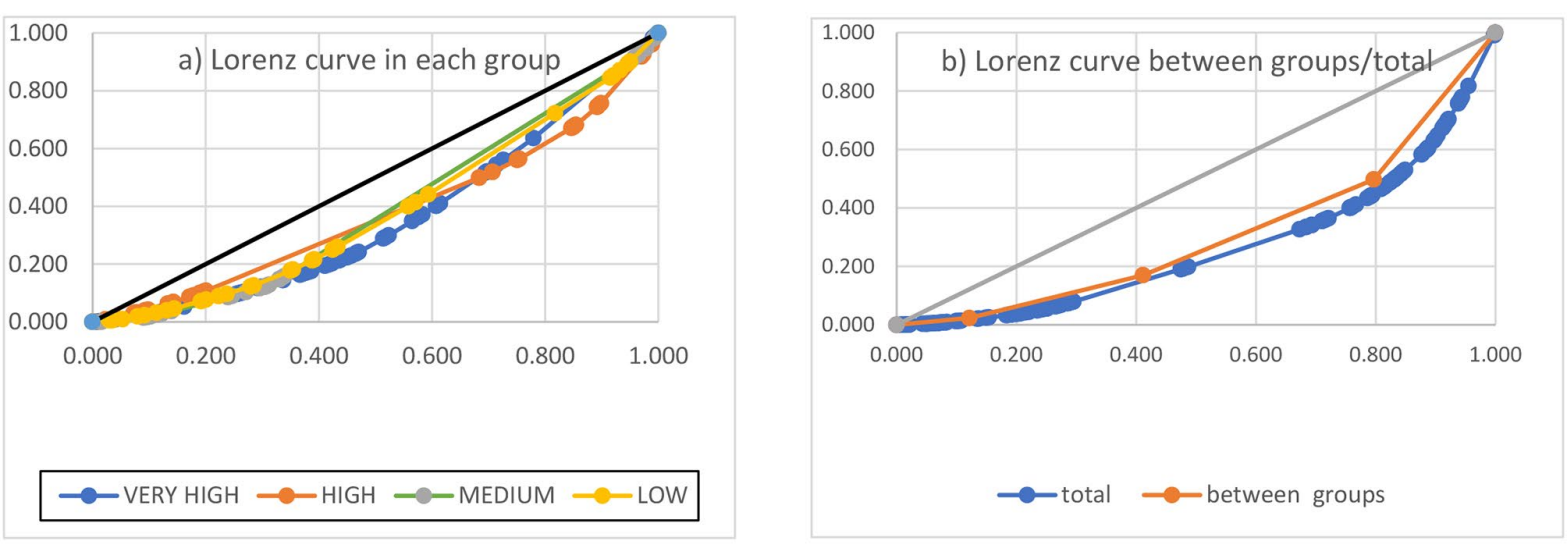
Lorenz curves for nurse distribution per population. **(a)** Lorenz curve in each HDI group country and **(b)** Lorenz curve between HDI groups and total world countries

Gini coefficient calculations did not show significant differences between countries in each of the four HDI groups. Gini coefficients of the countries in each group ranged from 0.217 to 0.283. The highest Gini coefficient (0.283) pertained to the very high HDI group. The Gini coefficient between the four-group nations amounted to 0.467, and the coefficient of the whole world countries was 0.667 (Table 2). Figure 3 presents the Lorenz curve of each group of nations, between each of them and the whole world.

**Table 2:** Gini coefficient of nurse’s distribution in the world

Gini Coefficient	In each group				Between Groups	All over the world
	very high HDI	High HDI	Medium HDI	Low HDI		
	0.283	0.248	0.217	0.233	0.467	0.667

## Discussion

This study aimed to review the inequality of nursing workforce distribution worldwide. Consistent with the UN’s classification, world countries are divided into four categories of very high, high, medium, and low HDI indices. This study presents several general facts.

First, there were an average of 38.6 nurses per 10,000 population in the world. There was a gross difference between countries of the HDI indices by the number of nurses to the population, with the very high HDI nations having the highest nurse/population ratio (95/10,000) and the low HDI nations with the least nurse/population ratio (7/10,000). Pareto diagram analysis also confirms this difference in nurse distribution and suggests that over 80% of the world’s nurses are under very high and high HDI groups. Different studies have also reported these results [4, 6, 16, 20]. The world’s nursing workforce will have risen by 8 million by 2030, with 70% of this growth being in medium and high-income countries [17]. Boniol et al.applied the Gini coefficient to describe the differences in nursing density to the population in 58 countries out of six regions. These researchers concluded that the highest inequality of nursing personnel distribution pertained to Africa [4]. There was an 11% inequality difference between regions with the highest and lowest nurse density. In their study, 13% of the population had access to 45% of nurses [4]. In a survey of 147 countries, Wharrad and Robinsonargued that the nurse/population varied from 3/1000 in central Africa to 16.4/1000 in the north of Europe [18]. One of the reasons behind the unequal distribution of nursing resources appears to be the migration of health workers from developing countries to developed ones. Evidence suggests that the number of health workers in low- and middle-income countries has dropped, which even may worsen. While the Philippines has the largest number of immigrant nurses abroad, there are 30,000 vacant nursing posts in the country. About 25.5% and 18.4% of nursing positions in Ghana and Malawi are vacant, respectively [19–21]. In this respect, factors such as a low capacity for medical training, poor living and working conditions, and inadequate salaries could drive health workers out of the country [2]. Thus, establishing training strategies that focus on the development of continuous professions, together with improving working conditions and sufficient salaries, appear to be critical factors to nurses’ staying at their job positions in less developed areas. Such measures will certainly improve the distribution of the nursing workforce across the world.

Second, most nurses in the world are female and 35–44 years old. Lu et al. stated that females under 35 years were the most of the nursing workforce in china [11]. In a study by Masoumi et al., 80% of nurses in the Fars province of Iran were females [22]. Gunn et al. also confirmed gender inequality among nurses in 22 countries [23]. Thus, it is critical to develop and implement executive strategies to improve and maintain the credibility of the nursing profession and to train competent nurses by focusing on creating equal health care opportunities for both women and men.

Third, using the Gini coefficient led to no significant difference between the nursing workforce distributions in each HDI group of the nations. Gini coefficient values of the nurse distribution based on the HDI groups varied from 0.217 to 0.283, showing a relative equality. While the level of inequality between the four HDI groups based on the Gini coefficient was 0.467, there was a relatively higher level of inequality across the world with a Gini coefficient of 0.667. Different studies have reviewed the unequal distribution of the nursing workforce in various countries. Most of these studies were in China. Zhou et al. reported while the nurses’ Gini coefficient improved from 0.420 to 0.267 between 1985 and 2011, but among health workers, the distribution of nurses had the highest inequality between urban and rural areas [24]. Chen et al. reported that the Gini coefficient of the nurse-to-10,000 people in urban areas was 0.48 [25]. Also, the Gini coefficient of nurses in the Beijing province was 0.28 [26]. Other studies using the Gini coefficient, show inequality among Chinese and Indian populations was 0.471 and 0.527, respectively [10].

Few studies have also addressed inequality in nursing workforce distribution in other countries. For example, in Poland, the Gini coefficient of nursing distribution varied from 0.28 to 0.30 from 2010 to 2017 [8]. Wiseman et al. reported the Gini coefficient of nurse distribution to be 0.412 in different provinces of Fiji and 0.077 for the intro-provincial areas [27]. There was inequality in the distribution of nursing employees in different parts of Cameroon using the Gini coefficient of 0.307 [6]. Likewise, in Brazil, nurses had the highest inequality compared to other employees from 1991 to 2005, with the poorest states suffering from the highest shortage of health workers. Hence, they face the highest inequality in the distribution of physicians and nurses [5]. The Gini coefficient of the nurses-to-population distribution in the provinces of Cambodia was 0.29 [28]. Inequality of nurse distribution in Tehran also was 0.228–0.315 from 2007 to 2013 using the Gini coefficient [29].The inequality of the nursing employee distribution in South Khorasan Province (Eastern Iran) using the Gini coefficient was 0.51 in 2018 [30]. By implementing workforce distribution reform policies, countries like Turkey have managed to reduce the Gini coefficient of nurse distribution from 0.2 to 2002 to 0.11 in 2016 (15). Ismail showed that the health resources based on the population size in the 18 states of Sudan were unequally distributed, and the Gini coefficient of nursing employees was 0.47 [7]. The findings of these studies confirmed the unequal distribution of nursing workforce in different regions and countries.

As stated, the inequality of human resources for healthcare services is a global concern. The WHO and other European commissions have taken major initiatives to deal with human resources challenges for healthcare services. In its 2004 report, the WHO explains that healthcare workers are integral parts of the healthcare systems. According to this report, continuous and updated studies are lacking in this regard. Also, emphasis has been put on improving human resources for healthcare systems within the international political agenda [8]. Therefore, policymakers should focus on the equitable distribution of health workforce at local, national, and regional levels. To this end, WHO should consider the geographical distribution of the nursing workforce within the regional health planning process for improved health resource allocation.

## Conclusion

Human resources play a major role in meeting the goals defined by the health system. This study focused on the equal distribution of the world’s nursing workforce. The geographical distribution of nurses was found to be disproportionate to the population distribution. Also, there was more inequality across world than in countries with different HDI groups. There are a number of gaps in our knowledge around the topic in this research. Hence, the authors recommend that Future researches could further examine the correlation between the human development index and the number of nurses in the country, the number and distribution of unregistered nurses in the world and also equality in the distribution of nurses in different countries based on their academic education level. The results of this study can be used in better planning and organization of human resources to improve the health care system. Based on the results of this study, WHO and UN are suggested to help their member countries in the redistribution of nurses in underprivileged areas such as AFRO and, if possible, provide the possibility of transferring nurses between countries in each of the six regions. In cases where it is impossible to transfer nurses between countries, WHO should try to train a larger number of nurses in those countries by providing educational aid to them. Moreover, WHO’s use of multi-professional health workers in cases where countries are facing a shortage of nurses would be beneficial.

## Data Availability

The datasets used and/or analyzed during the current study are available from the corresponding author on reasonable request.
